# Pharmacogenetics Biomarkers and Their Specific Role in Neoadjuvant Chemoradiotherapy Treatments: An Exploratory Study on Rectal Cancer Patients

**DOI:** 10.3390/ijms17091482

**Published:** 2016-09-05

**Authors:** Eva Dreussi, Erika Cecchin, Jerry Polesel, Vincenzo Canzonieri, Marco Agostini, Caterina Boso, Claudio Belluco, Angela Buonadonna, Sara Lonardi, Francesca Bergamo, Sara Gagno, Elena De Mattia, Salvatore Pucciarelli, Antonino De Paoli, Giuseppe Toffoli

**Affiliations:** 1 Experimental and Clinical Pharmacology, Centro di Riferimento Oncologico, National Cancer Institute, Aviano 33081, Italy; edreussi@cro.it (E.D.); ececchin@cro.it (E.C.); sgagno@cro.it (S.G.); edemattia@cro.it (E.D.M.); 2 Unit of Cancer Epidemiology, Centro di Riferimento Oncologico, National Cancer Institute, Aviano 33081, Italy; polesel@cro.it; 3 Pathology, Centro di Riferimento Oncologico, National Cancer Institute, Aviano 33081, Italy; vcanzonieri@cro.it; 4 First Surgical Clinic Section, Department of Surgical, Oncological and Gastroenterological Sciences, University of Padua, Padova 35128, Italy; m.agostini@unipd.it (M.A.); puc@unipd.it (S.P.); 5 Nano-Inspired Biomedicine Laboratory, Institute of Pediatric Research-Città della Speranza, Corso Stati Uniti 4, Padova 35127, Italy; 6 Department of Nanomedicine, The Methodist Hospital Research Institute, 6670 Bertner Avenue, Houston, TX 77030, USA; 7 Radiation Oncology, Istituto Oncologico Veneto—IRCCS, Padova 35128, Italy; caterina.boso@ioveneto.it; 8 Surgical Oncology, Centro di Riferimento Oncologico, National Cancer Institute, Aviano 33081, Italy; cbelluco@cro.it; 9 Medical Oncology B, Centro di Riferimento Oncologico, National Cancer Institute, Aviano 33801, Italy; abuonadonna@cro.it; 10 Medical Oncology 1, Istituto Oncologico Veneto—IRCCS, Padova 35128, Italy; sara.lonardi@ioveneto.it (S.L.); francesca.bergamo@ioveneto.it (F.B.); 11 Radiation Oncology, Centro di Riferimento Oncologico, National Cancer Institute, Aviano 33081, Italy; adepaoli@cro.it

**Keywords:** pharmacogenetics, locally advanced rectal cancer, chemoradiotherapy

## Abstract

*Background*: Pathological complete response (pCR) to neoadjuvant chemoradiotherapy (CRT) in locally advanced rectal cancer (LARC) is still ascribed to a minority of patients. A pathway based-approach could highlight the predictive role of germline single nucleotide polymorphisms (SNPs). The primary aim of this study was to define new predictive biomarkers considering treatment specificities. Secondary aim was to determine new potential predictive biomarkers independent from radiotherapy (RT) dosage and cotreatment with oxaliplatin. *Methods*: Thirty germ-line SNPs in twenty-one genes were selected according to a pathway-based approach. Genetic analyses were performed on 280 LARC patients who underwent fluoropyrimidine-based CRT. The potential predictive role of these SNPs in determining pathological tumor response was tested in Group 1 (94 patients undergoing also oxaliplatin), Group 2 (73 patients treated with high RT dosage), Group 3 (113 patients treated with standard RT dosage), and in the pooled population (280 patients). *Results*: Nine new predictive biomarkers were identified in the three groups. The most promising one was rs3136228-*MSH6* (*p* = 0.004) arising from Group 3. In the pooled population, rs1801133-*MTHFR* showed only a trend (*p* = 0.073). *Conclusion*: This exploratory study highlighted new potential predictive biomarkers of neoadjuvant CRT and underlined the importance to strictly define treatment peculiarities in pharmacogenetic analyses.

## 1. Introduction

The standard treatment of locally advanced rectal cancer (LARC) in Europe is represented by a multimodal approach. Patients undergo neoadjuvant radiotherapy (RT) or chemoradiotherapy (CRT) primarily based on fluoropyrimidines (5-fluorouracil (5-FU) or capecitabine), sometimes associated with platinum derivatives or monoclonal antibodies, followed by surgery and adjuvant treatment if needed.

Response to neoadjuvant therapy plays a pivotal importance in rectal cancer patients. This treatment aims at reducing the tumor mass leading to a more sparing surgery choice, reducing the risk of the side effects, such as urinary and sexual dysfunction, fecal urgency and incontinence [1]. Moreover, neoadjuvant therapy can affect patients’ prognosis, in terms of overall and disease-free survival, and has also an effect on the risk of distant metastases and on local recurrence control [2]. However, despite the big efforts spent to optimize response to neoadjuvant therapy, tumor downstaging/downsizing and pathological complete response (pCR) after neoadjuvant treatment are achieved by only a minority of patients, ranging from 10% to 35% [2,3,4].

The identification of reliable predictive biomarkers represents a pivotal need for optimizing cancer patients’ treatment, leading to fewer invasive surgical approaches in case of pCR, and to upfront surgery or intensified neoadjuvant treatment in patients with a lower possibility to achieve treatment response.

Many studies have already addressed this issue. One discipline with big potentialities in this field is represented by pharmacogenetics, which aims at defining genetic variants, called polymorphisms, that affect response to therapy in terms of both efficacy and risk to develop toxicities. The relevance of common variations in DNA sequences like nucleotides’ deletion/insertion, microsatellites, and single nucleotide polymorphisms (SNPs), is related to their possible implication in gene expression regulation or in their influence on protein activity, thus impacting pharmacokinetics and pharmacodynamics. In particular, germline variants exert a potential impact on the pharmacodynamics of drugs and on the body reaction to treatment [5], and their analyses have already highlighted new potential biomarkers to be translated into clinical practice like SNPs on *TYMS*, *XRCC1*, *EGFR*, and *MTHFR* [6]. As a consequence, once variations with reliable clinical value are identified, patients can be stratified according to their genotype and the most appropriate treatments can be selected. Thus, pharmacogenetics represents a discipline with big potentialities in the field of personalized medicine that for sure in the future will play a pivotal role in cancer patients’ management. Nonetheless, despite the introduction of some pharmacogenetic biomarkers in the international guidelines of some drugs, patients and clinicians are still waiting to reap the benefits potentially associated with genetics. Innovative and more precise study design could promote the introduction of genetics in the everyday clinical practice. In the case of the research of genetic predictive biomarkers of response to neoadjuvant treatment in LARC, a more comprehensive approach that takes into account the complex interplay of different cellular pathways could be the key to a better comprehension of this clinical problem. Moreover, more attention to treatment peculiarities is needed to properly compare results among different studies and to cope with the scarce clinical impact of pharmacogenetics in LARC patients’ management.

In light of these considerations and currently available literature data, we adopted a pathway-based approach in order to investigate the role of thirty SNPs in twenty-one genes involved in DNA repair system, proliferation and angiogenesis, and fluoropyrimidines response and toxicity. These cellular mechanisms orchestrate indeed in a direct way cellular response to treatment and tumor aggressiveness, thus determining patients’ response to neoadjuvant treatment. We have analyzed these SNPs on 280 LARC patients who underwent fluoropyrimidines-based CRT. These patients have been classified into three subgroups according to treatment characteristics, considering radiotherapy (RT) dosage and association with oxaliplatin. The primary aim of this study was to highlight potential predictive biomarkers according to the peculiarities of neoadjuvant treatment. The secondary aim was to identify potential predictive biomarkers suitable for the entire casistic.

We have identified nine potential predictive biomarkers from the analyses of patient subgroups. None of these SNPs was significantly associated with pCR in the pooled population, only a trend (*p* = 0.074) was highlighted for rs1801133-*MTHFR*.

Even if only preliminary and still not conclusive, these data underline the importance of these new predictive biomarkers in specific patient subgroups, thus highlighting the importance to strictly define treatment parameters when performing this kind of analysis. If validated in an external and larger validation group, they could represent an efficient tool for clinicians to personalize LARC patients’ treatment.

## 2. Results

### 2.1. Patients’ Characteristics and Clinical Outcome

We have collected from the medical records the following patients’ clinical and pathological data: age, gender, clinical tumor stage (cT), clinical nodal and metastasis stage (cN and cM, respectively), tumor distance from anal margin, neoadjuvant treatment parameters (RT dosage, fluoropyrimidines administration, concomitant platinum administration), date of diagnosis, surgery, end of RT, post-CRT pathologic T stage (ypT), kind of surgical intervention, intraoperative RT, recurrence, adjuvant treatment, and date of last follow-up/death. Moreover, the pathological response to neoadjuvant treatment according to Tumor Regression Grade (TRG) was collected for each patient. Patients’ characteristics are reported in Table 1.

### 2.2. Genotyping Results and Association of SNPs with pCR in the Three Groups of Patients

Genotyping analyses were successfully performed in our samples. The average genotype call rate was 97.24%, and 29 out of 34 assays had a call rate ≥96.00% (range: 92.14%–99.29%).

The association between genotypes and pCR was tested separately in the three subgroups of patients with multivariate analysis, adjusted for gender, age, RT dosage, distance of the tumor from the anal margin, concomitant platinum treatment, and interval between the end of RT and surgery (Table 2). Nine SNPs resulted significantly associated with pCR in at least one patient subgroup.

Two SNPs, rs2279744-*MDM2* and rs1801133-*MTHFR*, resulted significantly associated (*p* ≤ 0.05) to pCR in Group 1 according to a dominant model (*p* = 0.034, Adj-odds ratios (Adj-OR) = 0.24, 95% confidence interval (CI) = 0.07–0.90; *p* = 0.019, Adj-OR = 3.49, 95% CI = 1.23–9.91, respectively). In addition, according to an additive model, rs1130409-*APE1* was significantly associated to treatment response in Group 2 (*p* = 0.025, Adj-OR = 3.20, 95% CI = 1.16–8.84). The analyses in Group 3 highlighted six different potential predictive biomarkers. The most significant one (*p* < 0.01) was rs3136228-*MSH6*. According to the dominant model, bearing at least one variant allele reduced the risk not to obtain a pCR (*p* = 0.004, Adj-OR = 0.12, 95% CI = 0.03–0.51). Moreover, rs3213239-*XRCC1*, rs1799977-*MLH1*, and rs2010963-*VEGFa* played a potential strong predictive role (*p*-value < 0.025). Specifically, according to a dominant model, bearing at least one variant allele of rs3213239-*XRCC1* or of rs2010963-*VEGFa* increases the risk not to achieve a pCR (*p* = 0.020, Adj-OR = 3.24, 95% CI = 1.20–8.74; *p* = 0.022, Adj-OR = 3.14, 95% CI = 1.18–8.38, respectively). In addition, according to a recessive model, the rs1799977-*MLH1* is a protective factor (*p* = 0.026, Adj-OR = 0.23, 95% CI = 0.06–0.84). Finally, two other SNPs arose as predictive biomarkers according to a dominant model, even if their association is significant (*p* < 0.05) but not particularly strong considering the quite small sample size. Specifically, bearing at least one variant allele of rs3212986-*ERCC1* and rs12917-*MGMT* increases the risk not to achieve a pCR (*p* = 0.048, Adj-OR = 2.64, 95% CI = 1.01–6.90; *p* = 0.042, Adj-OR = 3.31, 95% CI = 1.05–10.45, respectively).

### 2.3. Association of SNPs with pCR in the Pooled Patients’ Population

Only one SNP, rs1801133-*MTHFR*, resulted significant in at least one group, showed a concordant genetic effect and compatible genetic models in the three subgroups. Thus, we have tested its predictive value in the pooled population. It did not maintain its significant predictive value, only a trend was observed (*p* = 0.073, Adj-OR = 1.46, 95% CI = 0.97–2.20).

## 3. Discussion

Different and unpredictable treatment response to neoadjuvant CRT in LARC represents a critical clinical problem. A not negligible percentage of patients did not achieve a pCR, thus rendering this treatment less effective in improving patients’ prognosis. The identification of patients who are at risk not to achieve a pCR could be the key to treatment optimization.

We aimed at determining new potential predictive biomarkers of neoadjuvant treatment. To this purpose, we considered the pCR, defined as TRG 1 (see Materials and Methods Section), as clinical endpoint, since its prognostic value is well known and corroborated also by our previous findings [7]. The key role exerted by this parameter has been demonstrated also in a recent meta-analysis that highlighted the superiority of TRG as surrogate prognostic marker compared to the tumor-, node-, metastasis TNM scale, one of the most widespread staging system of solid tumors [8].

We have focused our efforts on germline DNA because it could have a great clinical impact. Germline SNPs are indeed easily analyzable thanks to a blood draw. This is a minimal invasive procedure on patients, an important factor in the oncological setting.

**Figure 1:**
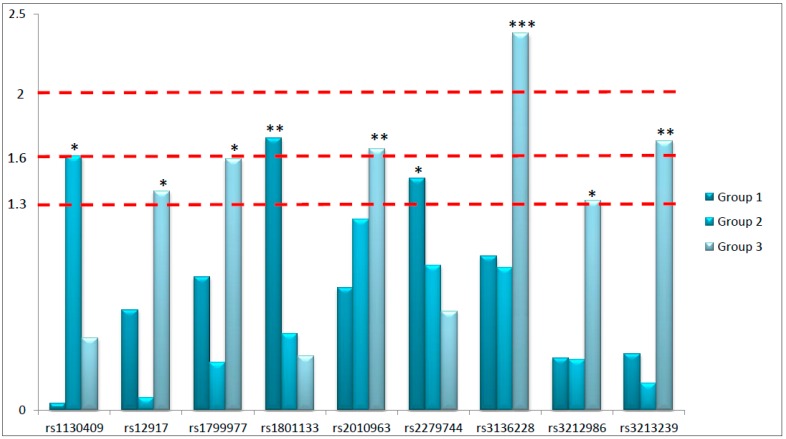
Graphical representation of the negative logarithms of the *p*-values (*Y*-axis) of the single nucleotide polymorphisms (SNPs) resulted significant in at least one group of patients. Predictive biomarkers were classified into those with the strongest association (*p* < 0.01), evidenced by three stars (***); those robustly associated (*p* < 0.025), with two stars (**); and those significantly associated with treatment response (*p* < 0.05), with one star (*). Dash lines were traced according to the selected thresholds of *p*-values.

The primary aim of this study was to highlight potential predictive biomarkers considering the peculiarities of the prescribed neoadjuvant treatment. For this purpose, we have analyzed thirty SNPs in twenty-one different genes involved in key pathways of response to RT and fluoropyrimidines-based chemotherapy in 280 LARC patients. In particular, we focused our attention on genetic variants located in genes involved in DNA repair pathways, proliferation and angiogenesis, and response to fluoropyrimidines. We have searched for their independent predictive role in predicting the complete pathological response to neoadjuvant therapy in three different patient subgroups defined according to treatment peculiarities. Globally, nine SNPs arose as potential predictive biomarkers of response to neoadjuvant treatment: six located in genes involved in DNA repair, two in genes controlling proliferation and angiogenesis, and one in a gene modulating fluoropyrimidines response. Considering the explorative nature of this study, we had coarsely classified the identified predictive biomarkers according to the obtained *p*-values in order to underline the strength of the associations (Figure 1). Specifically, we roughly divided the predictive biomarkers into those with the strongest association (*p* < 0.01), those robustly associated (*p* < 0.025), and those significantly associated (*p* < 0.05) with treatment response. Interestingly, from the analysis of Group 3 we obtained six out of nine significant predictive biomarkers, including the most significant one (rs3136228-*MSH6*), and two SNPs robustly associated with pathological response (rs3213239-*XRCC1* and rs2010963-*VEGFa*, *p* < 0.025). This is a really interesting point considering that Group 3 represents the standard treatment, based on fluoropyrimidines and standard dose of RT (5040 cGy).

The strongest association (*p* < 0.01) obtained in this study is represented by rs3136228-*MSH6*. The *MSH6* gene codifies for a factor mainly involved in mismatch repair (MMR) and in other kind of DNA repair mechanisms such as the response to double strand breaks (DSB). Specifically, MSH6 seems to be involved in non-homologous end joining pathway, that is activated in case of DNA breaks. Interestingly, in vitro MSH6 deficiency leads to an impaired DSB repair, with a consequent higher sensitivity to cell death induced by radiation exposure [9]. These data are further corroborated by analyses on cancer patients. Altered levels of proteins involved in MMR like MSH6, MLH1, and MSH2, have been observed after neoadjuvant treatment possibly as response to hypoxia and oxidative stress [10]. Very recently, the group of Huh analyzed the protein expression of MSH6 in biopsies and surgical specimens on 209 LARC patients undergoing preoperative CRT. It was observed a significant association between MSH6 levels in biopsies and overall survival and between MSH6 levels in surgical specimens and local recurrence, shedding light to the pivotal role exerted by MSH6 in this clinical setting. Nonetheless, they failed to find any predictive role of this factor [11], even if its involvement in RT response in other clinical settings, like brain tumors, has already been demonstrated [12]. The rs3136228-*MSH6* is located in the upstream region of the gene and seems to affect the binding with the transcription factor Sp1 [13], having a potential role in gene transcription and thus in protein levels and in MMR efficiency. Interestingly, this SNP has been associated with the toxic effects due to fluoropyrimidines and oxaliplatin in the FOLFOX regimen in metastatic CRC patients [14]. Thus, even if there are no previous associations between this SNP and neoadjuvant response in LARC, literature data about the role of this protein and of this genetic variant in various oncological settings seem to reinforce our finding. It is quite puzzling that this SNP resulted significant only in Group 3, which represents the standard treatment of LARC patients. In order to give some suggestions about this, we can consider the DNA repair mechanisms mainly activated in the three different settings. Patients belonging to Group 2 were treated with high dosage of RT. This treatment promotes primarily the formation of DNA strand breaks, which are predominantly recognized by homologous recombination and non-homologous end joining mechanisms, while MMR plays a marginal role in this case. This observation can support the lack of significance of rs3136228-*MSH6* in predicting tumor response in this subgroup. The lack of significance in Group 1 is trickier to interpret due to the central role of MMR in response to oxaliplatin. Nonetheless, we can suppose that oxaliplatin, fluoropyrimidines and RT act in a synergistic effect promoting more impacting damages in DNA. However, this result claims for further analyses in order to verify our hypothesis and to confirm the predictive value of this SNP.

Other biomarkers strongly associated with treatment response (*p* < 0.025) are rs3213239-*XRCC1*, rs2010963-*VEGFa*, rs1801133-*MTHFR*, and rs1130409-*APE1*.

Two biomarkers strongly associated with treatment response, rs3213239-*XRCC1* and rs2010963-*VEGFa*, arose from the analysis conducted in Group 3. The *XRCC1* gene codifies for a protein with a central role in base excision repair pathway, in DNA break repair, and DNA damages caused by oxidation. The importance of this gene in cellular physiology can be better conceived considering that *XRCC1* knock-out mice are embryonic lethal [15]. Therefore, genetic variants in this gene may exert an effect not only in cancer onset but also on treatment response. The rs3213239-*XRCC1* is an insertion/deletion polymorphism located in the upstream region of the gene. At the best of our knowledge, its biological function has not been defined. We were also not able to predict the function of the polymorphism through a bioinformatic approach. Nonetheless, we can suppose that this region could exert a regulatory role in gene transcription, thus having a potential impact on mRNA levels and on cell response to DNA damages. This potential biological function could be supported by literature data available at the moment about the role of this SNP in cancer. Specifically, it has been associated with the susceptibility to B-cell acute lymphoblastic leukaemia [16]. Our study is the first one, at the best of our knowledge, to address its potential role in predicting treatment response. We can hypothesize that this genetic variant impact XRCC1 expression levels, that ultimately have an effect on response to CRT. Another strong predictive biomarker in Group 3 is represented by rs2010963-*VEGFa*. This protein is strictly involved in angiogenesis, which exerts a pivotal role in determining RT response. Poor tumor oxygenation leads indeed to resistance to RT and can negatively affect also drug intratumoral accumulation. Moreover, angiogenesis is fundamental to promote cell proliferation and resistance to apoptosis, thus negatively impacting patients’ prognosis [17]. This is the rational of the introduction of bevacizumab together with RT in neoadjuvant treatment of LARC patients [18]. The rs2010963-*VEGFa* is located in the 5’UTR, region enriched of regulatory motifs, that accounts for the association of this SNP with VEGF serum levels [19]. This SNP has been classified as risk factor for many cancer types, as also highlighted by a meta-analysis [20]. This can sustain the result that we have obtained in our analysis. Interestingly, in Group 2 we observed a not significant trend between this SNP and treatment response, possibly highlighting the key role of this factor in determining patients’ response to RT and fluoropyrimidines, that was not observed in the case of oxaliplatin treatment. However, the result cannot be considered as conclusive.

Another robust predictive biomarker (*p* < 0.025) was rs1801133-*MTHFR*, that arose from the analyses conducted on Group 1. This gene has been selected for its central role in fluoropyrimidines response. It codifies for an enzyme, methylentetrahydrofolate reductase, that catalyzes the conversion of 5,10-methylentetrahydrofolate (5,10-MTHF) to 5-MTHF. Cellular levels of 5,10-MTHF can exacerbate the cytotoxicity exerted by 5-FU. The rs1801133-*MTHFR* is a missense SNP that causes a substitution of alanine to valine in position 222, thus inducing a 60% reduction in enzyme activity [21]. Many studies have already addressed the potential association between this SNP and patients’ response to fluoropyrimidines treatment, but the obtained data cannot be considered as conclusive. The potential clinical value of rs1801133-*MTHFR* has been specifically investigated in colorectal- and rectal cancer setting [22,23]. Our group has previously highlighted its role in predicting pathological tumor response to neoadjuvant treatment in a group of LARC patients [24]. Nonetheless, we cannot compare these two analyses because they differ from the kind of prescribed treatment and from the classification of patients’ response. As already stated, we can suppose that the effect of this SNP may be significant only for specific patients subpopulations, but this remains to be validated.

Finally, rs1130409-*APE1* is the only biomarker identified in the analysis of Group 2, characterized by high dose of RT. APE1 is a key factor of base excision repair pathway, that removes damaged single DNA bases and efficiently repair single-strand breaks extensively generated by radiation therapy. More in detail, this factor is the major nuclease involved in the recognition of apurinic/apyrimidinic sites in DNA, which can be formed by spontaneous hydrolysis, DNA damaging agents or by glycosylases. This kind of damage can be induced also by RT. Moreover, APE1 acts also as redox modulator regulating the activity of transcription factors as Nf-κB1, that ultimately plays a pivotal role in inflammation [25]. The involvement of APE1 in cancer response to treatment can be justified not only by a direct involvement of this factor in DNA repair but also considering its role in determining tumor microenvironment. Interestingly, some authors have suggested APE1 as new potential druggable target [26,27]. The rs1130409-*APE1* is a missense variant that causes an amminoacidic change in position 148: an aspartic acid is substituted by a glutamic acid. The SNP does not affect the activity of this nuclease, even if cells bearing the variant allele present a higher radiosensitivity [28,29]. The role of this SNP in predicting toxicities induced by radiation therapy has been already reported in lung cancer [30] even if, at the best of our knowledge, not previous significant associations between this SNP and treatment response in LARC patients have been reported. We can suppose that high RT dosage enhances the formation of single strand breaks and increases the oxidative stress, thus potentially justifying the significant association between rs1130409-*APE1* and treatment response obtained only in Group 2.

Ultimately, other SNPs with a potential predictive role evidenced by our analysis were rs3212986-*ERCC1*, rs1799977-*MLH1*, rs12917-*MGMT*, and rs2279744-*MDM2*, even if the observed associations seem not to be particularly strong.

The secondary aim of this study was to identify potential predictive biomarkers suitable for the entire group of patients. We had introduced this secondary aim in order to highlight biomarkers with a potential easier introduction in the clinical practice due to their less specificity to treatment peculiarities. To avoid the selection of false positive biomarkers, we compared the effect of the SNPs among the three different subgroups, selecting only those resulted significant in at least one subgroup, with a concordant genetic effect (increased or reduced possibility to achieve pCR) and compatible genetic models in the three subgroups. Only rs1801133-*MTHFR* satisfied these criteria, but the analysis in the pooled population evidenced only a trend in predicting treatment response (*p* = 0.073). It is quite perplexing that we did not find any biomarker suitable for the entire population, but we can ascribe this to the not homogeneous treatments included in this analysis. This study addresses the potential strong association of genetic biomarkers to specific neoadjuvant treatments administered to LARC patients. The specificity of the biological response to various insults can justify the observed differences among the new potential biomarkers identified in the three patient subgroups. The biomarkers potentially characterizing Group 1 are associated with fluoropyrimidines pathway (rs1801133-*MTHFR*) and with cell proliferation (rs2279744-*MDM2*), whereas those of Groups 2 and 3 belong mainly to DNA repair mechanisms (rs1130409-*APE1*, rs3213239-*XRCC1*, rs3136228-*MSH6*, rs3212986-*ERCC1*, rs1799977-*MLH1*, and rs12917-*MGMT*) and to angiogenesis (rs2010963-*VEGFa*). These data are hypothesis generating and suggest an important impact of treatment peculiarities in tumor response. This specificity is also underlined by the lack of a solid predictive biomarker that fits for the pooled population. Nonetheless, the explorative nature of this study cannot allow us to draw final conclusions. Performing the analysis on a validation group of patients is mandatory to clearly and ultimately define the potential predictive role of the identified genetic variants. It would be also of great potential clinical interest to investigate the prognostic value of these SNPs in terms of disease-free survival and overall survival in order to optimize LARC patients’ management.

We have to acknowledge that this study presents some limits. First of all, it is necessary to underline the small sample size of the analyzed subgroups, which cannot give the ultimate response to the need to identify strong biomarkers to be translated into the clinical practice. As already stated, these results are preliminary and only hypothesis generating. However, the performed multivariate analysis gave an additional value to the obtained results. In addition, a prioritization of our data according to the strength of the associations has been suggested in order to conceive them in a critical way. Moreover, as already stated, we have to underline that we did not have the possibility to validate these results in an external validation group of LARC patients, that is necessary to identify biomarkers of clinical interest. This is for sure one of the main limits of this study. Nonetheless, we believe that these preliminary results are of some interest because they are based on a numerous group of LARC patients with homogeneous clinical characteristics. Consequently, due to the explorative nature of this analysis, we hope that it could be considered as a starting point for future analyses. Furthermore, in some cases the biological function of the identified biomarkers has not been elucidated yet, thus rendering not possible the fully comprehension of the obtained results. Another potential limit is represented by the total independence of the results among the three subgroups, even if all patient underwent fluoropyrimidines-based CRT. On the one hand, this sustains our hypothesis about the central role of treatment peculiarities, such as association treatments and RT dosage. On the other hand, it is quite puzzling that none of the selected SNPs could be considered as potential predictive biomarker of response to neoadjuvant treatment suitable for the pooled population. Only a trend was observed for *MTHFR*, a central gene in fluoropyrimidines response. Analyses performed in larger populations could for sure be the key to solve these dilemmas. In addition, a more comprehensive approach that considers the joint effect of different SNPs and also of clinical parameters could pave the way to a more holistic comprehension of treatment response that barely can be considered as a monogenic trait due to the involvement of several cellular pathways. This strategy requires for sure larger groups of patients but could suggest a more efficient model to explain and predict treatment response.

## 4. Materials and Methods

### 4.1. Patients 

As previously described [7], from December 1993 to July 2011, 280 patients were enrolled by Centro di Riferimento Oncologico CRO-Aviano National Cancer Institute and by Istituto Oncologico Veneto IOV-IRCCS and First Surgical Clinical Section of Padova University, Padua, Northern Italy. Patients were considered eligible according to these criteria: histologically confirmed diagnosis of primary resectable LARC, confirmed absence of distant metastases, age ≥18 years, Caucasian ethnicity, stage of disease T3–T4 and N0–2, performance status (World Health Organization) 0–2, normal bone marrow, renal, and liver function. Patients were treated in neo-adjuvant setting with CRT based on fluoropyrimidines (either 5-FU or capecitabine) with or without oxaliplatin, combined with a dose of 5040 cGy or 5500 cGy of RT.

All procedures were reviewed and approved by the Ethical Committee of each participating institution, and all patients signed a written informed consent for research purposes. Specifically, patients were enrolled according to Phase III study on rectal cancer INTERACT-LEADER CRO-07-2006, approved by the Ethics committee CRO-Aviano on 20 January 2006, and according to the Study RF-2011-02349645 about the use of non invasive biomarkers on rectal cancer, 3533/AO/15 approved by the Ethics committee of Padua on 21 June 2016.

### 4.2. Patients’ Treatment, Response Evaluation, and Follow up

As already described elsewhere [31], patients underwent external beam RT with a 10–18 mega-Volt (MV) linear accelerator. A 3D-CRT was used in all patients. Patients were treated in prone position with full-bladder. A dedicated up-down table was used for patient immobilization and small bowel dislocation outside the target volume, as previously reported [32]. The primary tumor, the mesorectum, the posterior wall of the bladder and prostate/vagina, and the internal iliac nodes represented the clinical target volume (CTV).

Two different RT programs were performed, according to clinical trials ongoing in the considered period time. Specifically, in the first RT program patients were treated with a standard dose of 50.4 Gy/28 fractions: a consecutive boost of 50.4 Gy/3 fractions to the tumor and mesorectum was given following the CTV dose of 45.0 Gy/25 fractions, for a total dose of 50.4 Gy. In the second program, patients underwent a dose of 55.0 Gy/25 fractions: specifically, a concomitant boost of 10 Gy/10 fractions over 5 weeks, 2 times a week (1 Gy/fraction, 6 h interval between the two daily fractions), was delivered to the tumor and mesorectum during the CTV dose of 45 Gy fractions, for a total dose of 55 Gy. Fluoropyrimidines alone (5-FU 225 mg/m^2^/day intravenous continuous infusion for 5 weeks or capecitabine 1650 mg/m^2^ in two daily oral administrations for 5 weeks) was prescribed with 50.4 Gy or 55.0 Gy, whereas the capecitabine (1300 mg/m^2^) was administered with oxaliplatin (130 mg/m^2^ every 19 days) and concurrently standards RT dose of 50.4 Gy.

Standard pathological tumor staging of the surgical specimens was performed in accordance with the guidelines of the American Joint Committee on Cancer. Treatment efficacy was defined as TRG [33], and assessed as previously described [34]. Briefly, it is a 5-point histological scale based on the level of fibrosis and tumor cells in surgical specimen. Patients with TRG 1 have achieved a complete pathological response, whereas patients with TRG 2–5 still present viable cancer cells in surgical specimen. All patients were followed-up every 3 months for the first 2 years, every 6 months thereafter up to 5 years, and then yearly.

### 4.3. SNPs Selection and Genotyping Assays

**Figure 2:**
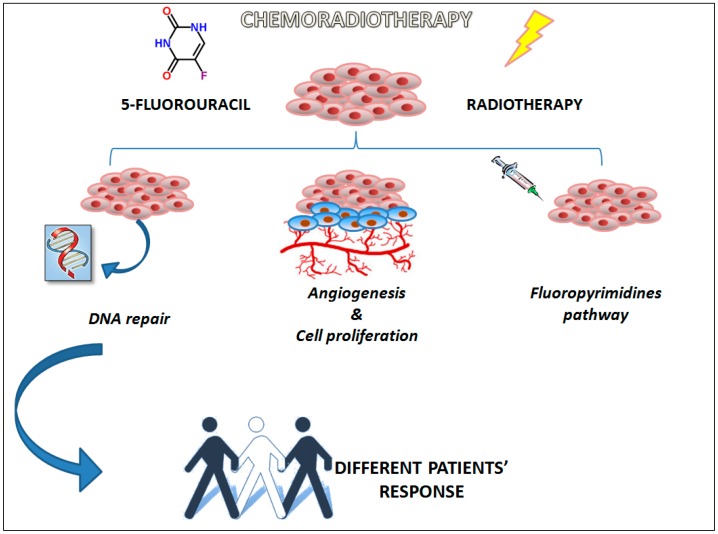
Pathway-based approach guiding this study. Response to fluoropyrimidine-based chemoradiotherapy is a complex phenomenon that is orchestrated by genes involved in many cellular mechanisms. Some key players are represented by DNA repair system, proliferation (blue cell) and angiogenesis, fluoropyrimidines response.

A set of thirty SNPs in twenty-one genes was selected. Specifically, according to literature data, we have chosen genes involved in pathways with a key role in chemoradiotherapy response (Figure 2). More in detail, we analyzed SNPs located in genes involved in different mechanisms regulating DNA repair (*XRCC1*, *XRCC3*, *MSH6*, *PARP1*, *OGG1*, *EXO1*, *ERCC2*, *ERCC1*, *MLH1*, *APEX1*, *MGMT*, *SOD2*, and *ATM*), in proliferation and angiogenesis (*TP53*, *MDM2*, *EGFR*, *EGF*, and *VEGFa*), and fluoropyrimidines response (*GSTP1*, *MTHFR*, and *TS*). The selection of the SNPs was based on literature data analysis. We have specifically focused our attention on the most promising genetic variants according to the literature data available at the time of the study design.

Genomic DNA of LARC patients was extracted from peripheral blood samples using the automated extractor BioRobot EZ1, in association with the Kit “EZ1 DNA Blood Kit 350 μL” (Qiagen SPA, Milano, Italy) and stored at +4 °C until the time of this study. Positive controls were included in the analyses. Pyrosequencing (Biotage, Uppsala, Sweden) was used for the analysis of rs3213239-*XRCC1*, rs1052133-*OGG1*, and rs2279744-*MDM2*. PCR amplifications were performed in a Eppendorf Mastercycler gradient, with TaqGold DNA polymerase (AB Applied Biosystem, Warrington, UK).

Pre-designed TaqMan SNP genotyping assays with the Applera TaqMan Universal Master mix were used on ABI 7900HT (AB Applied Biosystem, Foster City, CA, USA) according to the manufacturer’s instructions for the discrimination of the following SNPs: rs25487-*XRCC1*, rs25489-*XRCC1*, rs1799796-*XRCC3*, rs1799794-*XRCC3*, rs861539-*XRCC3*, rs3136228-*MSH6*, rs11136410-*PARP1*, rs4149963-*EXO1*, rs1799793-*ERCC2*, rs3212986-*ERCC1*, rs11615-*ERCC1*, rs1799977-*MLH1*, rs1130409-*APEX1*, rs12917-*MGMT*, rs1695-*GSTP1*, rs1138272-*GTSP1*, rs4880-*SOD2*, rs1642785-*TP53*, rs1801516-*ATM*, rs1801133-*MTHFR*, rs1801131-*MTHFR*, rs2227983-*EGFR*, rs4444903-*EGF*, rs2010963-*VEGFa*, and rs1570360-*VEGFa*. The gene copy number variations of *GSTT1* (Hs00767125_cn) and *GSTM1* (Hs00273142_cn) were analyzed with TaqMan technology assays.

Finally, rs17878362-*TP53* and rs34743033-*TS* were analyzed by PCR coupled with agarose gel electrophoresis.

Primers and PCR conditions are available upon request.

### 4.4. Statistics

Pathological tumor response to neo-adjuvant treatment was defined according to the TRG. This is an index that considers the amount of therapy-induced fibrosis in relation to residual tumor in surgical specimens. Complete responders (TRG 1: only fibrosis in surgical specimen) were compared to non-complete responders (TRG 2–5: increasing number of viable tumor cells in surgical specimen). We have performed this classification according to our previous analysis demonstrating the significant difference in patients with TRG 1 compared to patients with TRG 2–5 [7]. Patients were stratified in three different subgroups according to neoadjuvant treatment peculiarities, considering the concomitant administration of oxaliplatin and the RT dosages. In particular, Group 1 was formed by 94 patients treated with oxaliplatin, fluoropyrimidines, RT (5040 cGy); Group 2 by 73 patients treated with fluoropyrimidines and high dose RT (5500 cGy); and Group 3 by 113 patients treated with fluoropyrimidines and standard RT dosage (5040 cGy).

The association between genotypes and TRG was tested separately in the three groups of patients. Odds ratio (ORs) and 95% confidence interval (95% CI) were computed through logistic regression model, adjusting for gender, age, distance of the tumor from the anal margin, platinum treatment, RT dose, and time between the end of RT and surgery. Dominant, recessive, and additive genetic models were considered for each genotype combining heterozygous and homozygous genotypes. The best fitting genetic model was selected according to the Wald *X*^2^-test.

Polymorphisms resulting significant in at least one group, showing a concordant genetic effect, and with a “compatible” genetic model in the three groups were further investigated in the entire population. A “compatible” genetic model was considered the combination of an additive model in one group, with either a dominant or recessive model in the other group.

Logistic regression was performed using SAS 9.2 (SAS Institute Inc., Cary, NC, USA).

## 5. Conclusions

To conclude, this study highlighted the potential predictive key role of nine SNPs involved in key pathways of CRT response in three different LARC patient subgroups defined according to the peculiarities of neoadjuvant treatment. The strongest association (*p* < 0.01) was highlighted for rs3136228-*MSH6* in the patient subgroup undergoing standard treatment (fluoropyrimidines combined with a dose of 5040 cGy of radiotherapy). In the same subgroup, we identified two additionally strong biomarkers (*p* < 0.025), rs3213239-*XRCC1* and rs2010963-*VEGFa*. Moreover, other strong biomarkers (*p* < 0.025) arose in the group also undergoing oxaliplatin treatment, rs1801133-*MTHFR*, and in the group treated with high dose of RT, rs1130409-*APE1*. We did not find any significant association between genetic variants and pathological response to neoadjuvant treatment in the pooled population, only a trend was observed for rs1801133-*MTHFR*.

These data, even if only preliminary and hypothesis generating, address the great potentialities of genetic analyses performed on germline DNA and the importance of treatment peculiarities in determining new predictive biomarkers. If validated with further analyses performed in an external group of LARC patients with homogeneous clinical parameters, this study could lead to the introduction in the clinical practice of biomarkers to optimize patients’ treatment.

## Figures and Tables

**Table 1:** Demographic and clinical characteristics of locally advanced rectal cancer (LARC) patients analyzed in this study.

Demographic and Clinical Characteristics		
Parameters	Cases *N*	Cases %
Total	280	
Gender		
Males	191	68.21%
Females	89	31.79%
Age, years (Average (median, range))	61 (62; 20–82)	
Tumor distance from the anal verge (cm)		
≤7	189	67.50%
8–11	88	31.43%
≥12	3	1.07%
Treatment		
*Fluoropyrimidine administration*		
Bolus	15	5.36%
Continuous infusion	98	35.00%
Per os	155	55.36%
Not available	12	4.29%
*Tumor regression grade (TRG)*		
1	78	27.86%
2	58	20.71%
3	90	32.14%
4	43	15.36%
5	11	3.93%

**Table 2:** Odds ratios (OR) and 95% confidence interval (95% CI) for Tumor Regression Grade (TRG) 2–5 vs. TRG 1 in rectal cancer, according to gene polymorphism and treatment. All patients were treated with fluoropyrimidines-based chemoradiotherapy (CRT). Only single nucleotide polymorphisms (SNPs) resulted significant in at least one subgroup are reported. Statistically significant associations are in bold. Group 1: 94 patients treated with oxaliplatin, fluoropyrimidines, standard dose of radiotherapy (RT) (5040 cGy); Group 2: 73 patients treated with fluoropyrimidines and high dosage of RT (5500 cGy); Group 3: 113 patients treated with fluoropyrimidines and standard dose of RT (5040 cGy). All. ch.: allelic change; M: genetic model; R: recessive; A: additive; D: dominant.

Gene	SNP	All. ch.	Group 1			Group 2			Group 3		
			M	OR (95% CI)	*p* ^a^	M	OR (95% CI)	*p* ^a^	M	OR (95% CI)	*p* ^a^
*XRCC1*	rs3213239	I>D	D	1.56 (0.50–4.89)	0.443	R	1.57 (0.19–12.88)	0.676	**D**	**3.24 (1.20–8.74)**	**0.020**
*MSH6*	rs3136228	T>G	R	3.87 (0.75–20.01)	0.107	R	0.19 (0.02–1.60)	0.126	**D**	**0.12 (0.03–0.51)**	**0.004**
*ERCC1*	rs3212986	G>T	A	1.37 (0.59–3.18)	0.468	R	0.50 (0.07–3.40)	0.478	**D**	**2.64 (1.01–6.90)**	**0.048**
*MLH1*	rs1799977	A>G	D	0.44 (0.14–1.33)	0.144	R	0.48 (0.06–4.06)	0.498	**R**	**0.23 (0.06–0.84)**	**0.026**
*APE1*	rs1130409	T>G	D	0.94 (0.32–2.72)	0.906	A	**3.20 (1.16–8.84)**	**0.025**	R	0.53 (0.14–2.01)	0.350
*MGMT*	rs12917	C>T	D	0.51 (0.17–1.55)	0.232	A	1.15 (0.33–3.95)	0.831	**D**	**3.31 (1.05–10.45)**	**0.042**
*MDM2*	rs2279744	T>G	**D**	**0.24 (0.07–0.90)**	**0.034**	A	0.45 (0.16–1.24)	0.122	R	0.41 (0.10–1.79)	0.237
*MTHFR*	rs1801133 *	C>T	**D**	**3.49 (1.23–9.91)**	**0.019**	A	1.59 (0.63–4.03)	0.330	D	1.64 (0.45–5.93)	0.454
*VEGFa*	rs2010963	G>C	R	0.39 (0.10–1.50)	0.169	D	0.21 (0.04–1.09)	0.063	**D**	**3.14 (1.18–8.38)**	**0.022**

^a^ Adjusted for gender, age, RT dose, distance from anal margin, platinum-based chemotherapy, and time between radiation therapy and surgery; * Polymorphism resulting significant in at least one group, showing a concordant genetic effect and “compatible” genetic models in the three groups.
